# An engineered lipid remodeling system using a galactolipid synthase promoter during phosphate starvation enhances oil accumulation in plants

**DOI:** 10.3389/fpls.2015.00664

**Published:** 2015-08-31

**Authors:** Mie Shimojima, Yuka Madoka, Ryota Fujiwara, Masato Murakawa, Yushi Yoshitake, Keiko Ikeda, Ryota Koizumi, Keiji Endo, Katsuya Ozaki, Hiroyuki Ohta

**Affiliations:** ^1^Graduate School of Bioscience and Biotechnology, Tokyo Institute of TechnologyYokohama, Japan; ^2^Technical Department, Biomaterial Analysis Center, Tokyo Institute of TechnologyYokohama, Japan; ^3^Biological Science Laboratories, Kao CorporationTochigi, Japan; ^4^Core Research for Evolutional Science and Technology, Japan Science and Technology AgencyTokyo, Japan; ^5^Earth-Life Science Institute, Tokyo Institute of TechnologyTokyo, Japan

**Keywords:** monogalactosyldiacylglycerol, galactolipid, promoter, triacylglycerol, phosphate starvation, starch

## Abstract

Inorganic phosphate (Pi) depletion is a serious problem for plant growth. Membrane lipid remodeling is a defense mechanism that plants use to survive Pi-depleted conditions. During Pi starvation, phospholipids are degraded to supply Pi for other essential biological processes, whereas galactolipid synthesis in plastids is up-regulated via the transcriptional activation of *monogalactosyldiacylglycerol synthase 3* (*MGD3*). Thus, the produced galactolipids are transferred to extraplastidial membranes to substitute for phospholipids. We found that, Pi starvation induced oil accumulation in the vegetative tissues of various seed plants without activating the transcription of enzymes involved in the later steps of triacylglycerol (TAG) biosynthesis. Moreover, the *Arabidopsis* starchless phosphoglucomutase mutant, *pgm-1*, accumulated higher TAG levels than did wild-type plants under Pi-depleted conditions. We generated transgenic plants that expressed a key gene involved in TAG synthesis using the Pi deficiency–responsive *MGD3* promoter in wild-type and *pgm-1* backgrounds. During Pi starvation, the transgenic plants accumulated higher TAG amounts compared with the non-transgenic plants, suggesting that the Pi deficiency–responsive promoter of galactolipid synthase in plastids may be useful for producing transgenic plants that accumulate more oil under Pi-depleted conditions.

## Introduction

Plants possess various mechanisms, including membrane lipid remodeling (Essigmann et al., 1998; Härtel et al., 2000; Andersson et al., 2003, 2005; Jouhet et al., 2004; Nakamura, 2013; Shimojima et al., 2013), to adapt to inorganic phosphate (Pi)-limited conditions, which are distinct from those related to nitrogen (N)-limited conditions. During Pi deficiency, phospholipids in the biological membranes are degraded and the phosphorus generated via phospholipid breakdown is used for other essential biological processes in the cell.

To compensate for the lack of phospholipids in the membranes under Pi-limited conditions, galactolipid synthesis in the outer envelope membranes of plastids is up-regulated (Awai et al., 2001; Kobayashi et al., 2004). Plants have two species of galactolipids: monogalactosyldiacylglycerol (MGDG) and digalactosyldiacylglycerol (DGDG). Under normal growth conditions, both MGDG and DGDG are produced and remain in plastids as components of the thylakoid membrane (Benning and Ohta, 2005; Shimojima and Ohta, 2011). However, under Pi-depleted conditions the amount of DGDG doubles and is exported to the extraplastidial membranes by a yet unknown mechanism to substitute for the major phospholipid, phosphatidylcholine. In plastids, DGDG is mainly produced by an additional transfer of a galactose moiety to MGDG by DGDG synthases (DGD1 and DGD2) on the outer envelope membrane (Härtel et al., 2000; Kelly and Dörmann, 2002; Kelly et al., 2003). A previous analysis of an *Arabidopsis* mutant clearly showed that MGDG synthesis on the outer envelope membrane performed by type B MGDG synthases MGD2 and MGD3 has an important role in increasing the DGDG content during Pi depletion (Kobayashi et al., 2009). An *Arabidopsis mgd3* knock-out mutant showed a severe growth defect during Pi depletion, whereas an *mgd2* knock-out did not show a significant growth difference compared with wild type (WT; Kobayashi et al., 2009). Thus, between the two isoforms of type B MGDG synthase, MGD3 is predominantly involved in lipid remodeling during Pi starvation.

Type B MGDG synthase genes are widely conserved in seed plant genomes, suggesting that the enhancement of galactolipid synthesis under Pi starvation have been widely conserved in higher plants for adaptation to Pi-poor environments (Russo et al., 2007; Tjellström et al., 2008; Lambers et al., 2012; Yuzawa et al., 2012). Based on these previous findings, we hypothesized that a *MGD3* promoter might be useful for efficiently expressing introduced genes in the shoots and roots of plants in response to Pi starvation.

Plant storage lipids, triacylglycerols (TAGs), can be used as feedstock for the production of biodiesel or highly valuable fatty acids (Durrett et al., 2008; Dyer and Mullen, 2008; Riediger et al., 2009; Lu et al., 2011). However, most plant TAGs are synthesized and stored in seeds, which constitute a small portion of the total plant biomass. Although, TAGs are also synthesized in non-seed and vegetative tissues, such as leaves, the amount of TAG in vegetative tissues is usually very low (Chapman and Ohlrogge, 2012; Chapman et al., 2013). Many techniques have been used to increase TAG levels in vegetative tissues (Chapman and Ohlrogge, 2012; Chapman et al., 2013), and those approaches were based on knowledge obtained from TAG synthesis and breakdown in seeds. Most experiments involved ectopically overexpressing genes involved in TAG synthesis and knocking down/out genes involved in TAG breakdown. The transcription factors *LEAFY COTYLEDON1* and 2 (*LEC1* and *2*) are involved in seed maturation and TAG biosynthesis, respectively (Santos Mendoza et al., 2005; Mu et al., 2008). In *Arabidopsis*, the overexpression of *LEC1* or *LEC2* in WT plants and of *LEC2* in the fatty acid–breakdown mutant *COMATOSE* leads to TAG accumulation in vegetative tissues (Santos Mendoza et al., 2005; Mu et al., 2008; Slocombe et al., 2009; Kim et al., 2015). Transgenic tobacco that overexpresses *DIACYLGLYCEROL ACYLTRANSFERASE 1* (*DGAT1*) and *LEC2* also accumulates TAG in its leaves (Andrianov et al., 2010). Sanjaya et al. (2011) elevated TAG levels in vegetative tissues by (i) suppressing *APS1*, which encodes the small subunit of ADP-glucose pyrophosphorylase, which catalyzes the first step of starch biosynthesis, producing a starchless mutant and by (ii) overexpressing the transcription factor *WRINKLED1* (*WRI1*), which regulates TAG synthesis in *Arabidopsis* seeds and shoots. The ectopic overexpression of *Chlamydomonas reinhardtii* DGAT in *Arabidopsis* also elevates TAG levels in leaves (Sanjaya et al., 2013). The *Arabidopsis* COMPARATIVE GENE IDENTIFICATION-58 (CGI-58) homolog controls TAG breakdown exclusively in vegetative tissues by interacting with PEROXISOMAL ABC-TRANSPORTER 1 (PXA1), and the knockout mutant accumulates higher TAG levels in vegetative tissues compared with WT plants (James et al., 2010; Park et al., 2013). TAG levels in *Arabidopsis* vegetative tissues are also increased when *DGAT1* and *WRI1* are constitutively overexpressed in *sdp1* knockout mutant plants, which are defective in TAG breakdown under nutrient-sufficient conditions (Kelly et al., 2013). The overexpression of *PHOSPHOLIPID:DIACYLGLYCEROL ACYLTRANSFERASE 1* (*PDAT1*) with oleosin in *Arabidopsis* is also effective for enhancing TAG levels in leaves (Fan et al., 2013). Recently, Vanhercke et al. (2014) succeeded in producing transgenic *Nicotiana tabacum* (tobacco) in which TAG comprised >15% of the leaf dry weight by co-expressing three genes, *oleosin, DGAT1*, and *WRI1*, without severely affecting plant development. Thus, the previous reports clearly showed that genetically engineered vegetative tissues have the potential to store relatively high levels of TAGs.

Here, as an application of the lipid remodeling system during Pi starvation, we produced transgenic *Arabidopsis* plants that express high levels of TAG synthesis genes under the control of the *MGD3* promoter. We analyzed the effects of this approach on plant growth and the TAG content in vegetative tissues to evaluate its efficiency in producing more oil in plant vegetative tissues.

## Results

### Pi depletion increases TAG levels in *Arabidopsis* WT plants

**Figure 1:**
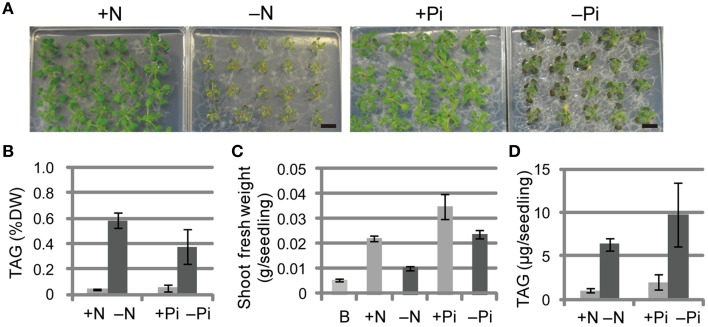
**Growth phenotypes and TAG accumulation in shoots of WT *Arabidopsis* plants grown under Pi- or N-depleted conditions**. WT plants (10 d old) were transferred to MS agar containing 1% (w/v) sucrose with (+Pi; 1 mM) or without (−Pi; 0 mM) Pi for 10 d, or with (+N; 3.5 mM) or without (−N; 0 mM) N for 7 d. **(A)** Growth under Pi-depleted or N-depleted conditions. Bars = 1 cm. **(B)** TAG levels in shoots under Pi-depleted or N-depleted conditions. DW, dry weight. **(C)** Shoot fresh weight under Pi-depleted or N-depleted conditions. B, before transfer to Pi- or N-depleted conditions. **(D)** TAG content in shoots per seedling under N- or Pi-depleted conditions. Data are the means ± SD from three independent experiments.

We first compared the phenotypes and TAG levels of WT plants grown under N-depleted and Pi-depleted conditions (Figure 1). The seedlings grown under N-depleted conditions were relatively more chlorotic than seedlings grown under Pi-depleted conditions (Figure 1A), and the TAG levels in N-depleted plants after 7 d were 1.5-fold higher than in the Pi-depleted plants after 10 d (Figure 1B), suggesting that the higher accumulation of TAG during N depletion was a consequence of the rapid breakdown of photosynthetic membranes. Consistent with the phenotypes shown in Figure 1A, WT seedlings grown under N-depleted conditions for 7 d were very small, and their shoot fresh weight was half that of plants grown under N-sufficient or Pi-depleted conditions (Figure 1C). The TAG content per seedling in WT shoots under Pi-depleted conditions was ~1.5-fold higher than that of WT shoots under N-depleted conditions (Figure 1D).

### Pi limitation induces TAG accumulation in vegetative tissues of various seed plants

**Figure 2:**
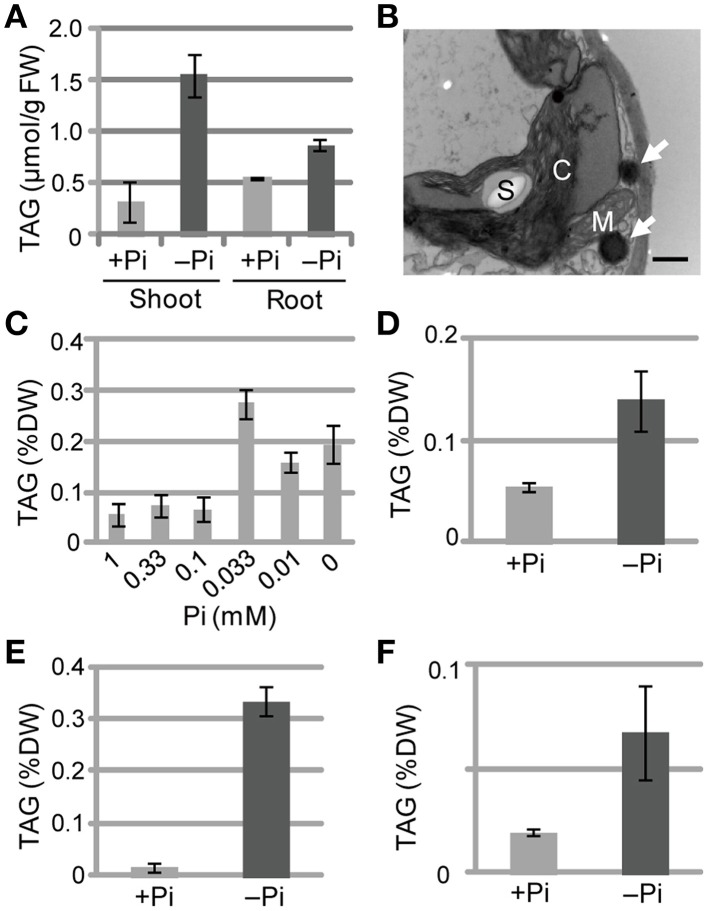
**TAG accumulation in WT plants under Pi-depleted growth conditions**. **(A,B)** WT *Arabidopsis* seedlings (10 d old) were transferred to MS agar containing 1% (w/v) sucrose and 0 mM (–Pi) or 1.0 mM (+Pi) Pi and were grown for 10 d. **(A)** TAG content in shoots and roots of WT *Arabidopsis* plants. FW, fresh weight. **(B)** Electron microscopy of WT *Arabidopsis* leaves. White arrows, oil droplets; C, chloroplast; M, mitochondrion; S, starch. Bar = 0.5 μm. **(C)** Effect of Pi concentration on shoot TAG levels in WT *Arabidopsis* plants. WT *Arabidopsis* seedlings (10 d old) were transferred to MS agar containing 1% (w/v) sucrose and various concentrations of Pi and were grown for 10 d. DW, dry weight. **(D)** TAG levels in shoots of tomato (*Solanum lycopersicum* L.) plants grown for 8 d with 1 mM Pi followed by 28 d with (+) or without (–) Pi. **(E)** Tobacco (*Nicotiana tabacum*) plants grown for 10 d with Pi followed by 21 d with (+) or without (–) Pi. **(F)** Barnyard grass (*Echinochloa crus-galli*) plants grown for 8 d with Pi followed by 28 d with (+) or without (–) Pi. Data are the mean ± SD from three independent experiments.

We also measured TAG levels in the roots of WT *Arabidopsis* plants grown under Pi-sufficient and Pi-depleted conditions. TAG levels in WT plants grown under Pi-depleted conditions were 5- to 6-fold higher in shoots and 1.5- to 2-fold higher in roots compared with those of plants grown under Pi-sufficient conditions (Figure 2A). It should be noted that we have also presented additional data here for shoot TAG levels for comparison with root TAG levels.

We used electron microscopy to assess the impact of low Pi availability on the accumulation of oil droplets in leaf mesophyll cells. Pi starvation resulted in many large starch granules within chloroplasts and oil droplets outside of chloroplasts, whereas no oil droplets were observed inside chloroplasts under these experimental conditions (Figure 2B; Supplementary Figure 1). At Pi levels between 0.1 and 1 mM, TAG levels were similar in plant leaves; however, when the Pi level was < 0.1 mM the TAG levels increased, with 0.033 mM Pi producing the highest TAG level (Figure 2C). Thus, a Pi concentration of ≤0.033 mM was required to observe the low Pi–dependent TAG accumulation in leaves. We also analyzed the TAG levels of other plant species under Pi-depleted conditions. Increased TAG levels upon Pi starvation also occurred in tomato (*Solanum lycopersicum* L.; Figure 2D; Supplementary Figure 2A), tobacco (*N. tabacum*; Figure 2E), and barnyard grass (*Echinochloa crus-galli*; Figure 2F; Supplementary Figure 2B), suggesting that the phenomenon is widely conserved among seed plants, including monocots.

### The vegetative tissues of the starchless phosphoglucomutase mutant *pgm-1* accumulate higher levels of TAGs under Pi-depleted conditions

**Figure 3:**
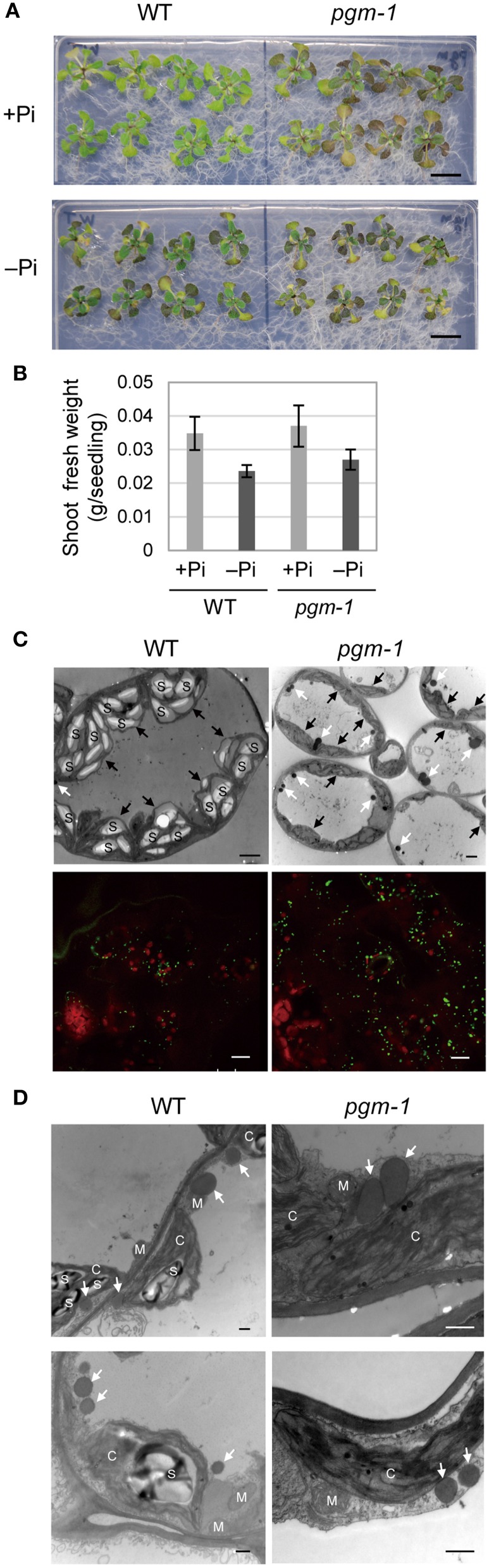
**Growth phenotypes and TAG accumulation in leaves of WT and starchless mutant *pgm-1 Arabidopsis* plants**. WT and *pgm-1* plants (10 d old) were transferred to MS agar containing 1% (w/v) sucrose and 0 mM (−Pi) or 1 mM (+Pi) Pi and were grown for 10 d. **(A)** Growth under Pi-sufficient (+Pi) or Pi-depleted (–Pi) conditions. Bars = 1.0 cm. **(B)** Shoot fresh weight of seedlings grown under Pi-sufficient and Pi-depleted conditions. **(C)** Oil droplets in leaf mesophyll cells under Pi-depleted conditions. Electron microscopy of leaf mesophyll cells (upper panels; white arrows, oil droplets; black arrows, chloroplasts; S, starch) and representative confocal fluorescence micrographs of leaves (lower panels) showing chloroplasts (red) and oil droplets stained with Nile red (green). Bars = 2 μm in upper images and 10 μm in lower images. **(D)** Electron microscopy of leaf mesophyll cells in WT and *pgm-1* plants grown under Pi-depleted conditions. White arrows indicate oil droplets. S, starch; M, mitochondrion; C, chloroplast. Bars = 0.5 μm.

Under Pi-depleted growth conditions, starch accumulates in leaf chloroplasts (Nielsen et al., 1998). Under nutrient-sufficient growth conditions, mutant *Arabidopsis* plants with low starch levels accumulate more TAGs in their vegetative tissues than WT plants (Sanjaya et al., 2011). To test whether the same pool of carbon sources was used for starch and oil synthesis in leaves under Pi-depleted conditions, we examined *Arabidopsis pgm-1* mutants, which lack almost all of the transitory starch in leaves because of a point mutation in the plastidic *phosphoglucomutase* gene (Caspar et al., 1985; Periappuram et al., 2000). Although, the shoots of *pgm-1* plants accumulated more anthocyanin than did WT plants under both Pi-sufficient and Pi-depleted conditions, their fresh weights were similar under both conditions (Figures 3A,B).

Lipid droplets in leaf mesophyll cells of WT and *pgm-1* plants grown under Pi-depleted conditions were visualized using electron microscopy (Figure 3C, upper panels) and also with a neutral lipid–selective fluorescent dye, Nile red (Figure 3C, lower panels, green). The number of lipid droplets was higher in *pgm-1* than in WT plants, and, based on electron microscopy observations, these lipid droplets were likely to be located outside of the chloroplasts (Figure 3D).

### Pi starvation–induced TAG accumulation occurs without the transcriptional activation of the key steps in TAG biosynthesis

**Figure 4:**
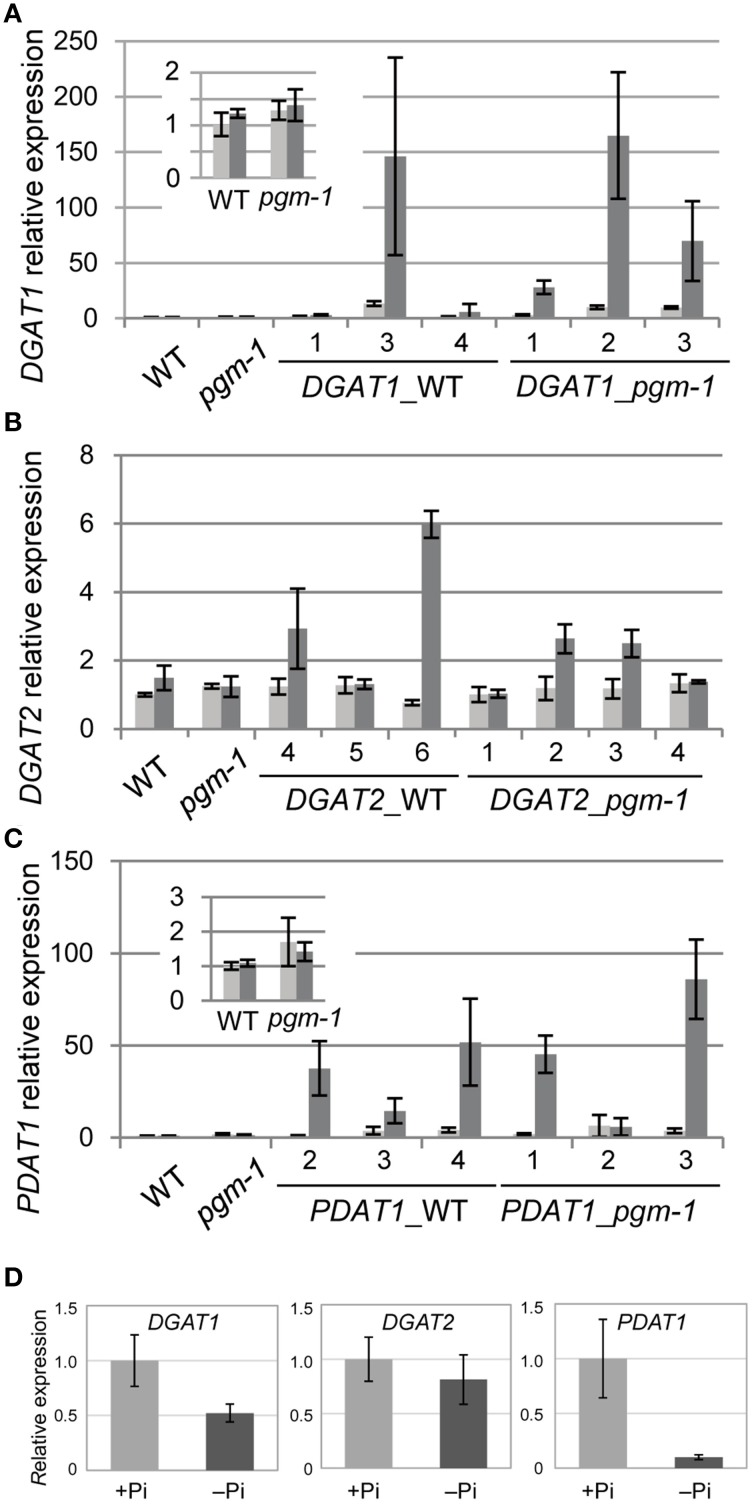
**Quantitative reverse transcription-PCR analysis of *DGAT1*, *DGAT2*, and *PDAT1* expression in WT, *pgm-1*, and transgenic *Arabidopsis* plants under Pi-sufficient or Pi-depleted conditions**. Seedlings (10 d old) of transgenic lines were transferred to MS medium containing 1% (w/v) sucrose, and 0 mM Pi (dark gray bars) or 1 mM Pi (light gray bars) and were grown for 10 d. **(A)** *DGAT1*, **(B)** *DGAT2*, and **(C)** *PDAT1* mRNA levels in shoots of WT, *pgm-1*, and transgenic lines in the WT and *pgm-1* backgrounds are shown. Line numbers are indicated for each transgenic strain. The expression level of each gene is relative to that in WT under Pi-sufficient conditions. Data are the mean ± SD from three independent experiments. **(D)** mRNA levels of *DGAT1, DGAT2*, and *PDAT1* in roots of WT plants under Pi-sufficient (+Pi) and Pi-depleted (–Pi) conditions. The expression level of each gene is relative to that in WT under Pi-sufficient conditions. Data are the mean ± SD from three independent experiments.

TAG accumulation during senescence is related to the transcriptional up-regulation of *DGAT1* (Kaup et al., 2002). TAG accumulation during N starvation occurs with the concomitant induction of the genes involved in TAG synthesis and accumulation, such as *DGAT1* and *OLEOSIN1* (Yang et al., 2011). Thus, we analyzed the expression of the key TAG biosynthetic genes *DGAT1, DGAT2*, and *PDAT1* in WT and *pgm-1* plants under Pi-sufficient and Pi-depleted conditions (Figures 4A–C). Distinct from TAG accumulation during senescence or N starvation, the high TAG accumulation in WT and *pgm-1* plants under Pi-depleted conditions (Figure 3C) did not correlate with the transcriptional up-regulation of these genes (Figures 4A–C). The expression levels of these genes in WT roots under Pi-sufficient and Pi-depleted conditions were also analyzed and were clearly shown to be unchanged or decreased during Pi starvation (Figure 4D). These results suggested that the overexpression of these genes under Pi-depleted conditions might further increase TAG levels in the leaves and roots of WT and *pgm-1* mutants.

### Pi starvation–induced overexpression of *DGAT1, DGAT2*, or *PDAT1* does not affect plant biomass

Previously, our group analyzed the promoter region of *Arabidopsis MGD3* (*ProMGD3*), which encodes a key enzyme in membrane lipid remodeling under Pi-depleted conditions. We showed a significant increase in *MGD3* expression levels in shoots and roots of WT plants upon Pi starvation (Awai et al., 2001; Kobayashi et al., 2004). To express TAG synthesis genes under Pi starvation, we produced transgenic *Arabidopsis* plants in WT and *pgm-1* backgrounds harboring the construct *ProMGD3:DGAT1* (designated as *DGAT1*_WT and *DGAT1_pgm-1*, respectively), *ProMGD3:DGAT2* (designated as *DGAT2*_WT and *DGAT2_pgm-1*, respectively), or *ProMGD3:PDAT1* (designated as *PDAT1*_WT and *PDAT1_pgm-1*, respectively). In shoots, compared with WT or *pgm-1* plants, *DGAT1* expression in *DGAT1*_WT line 3 and *DGAT1_pgm-1* line 2 were markedly higher under Pi-sufficient (~10- to 14-fold higher) and Pi-depleted (~140- to 170-fold higher) conditions (Figure 4A). In transgenic plants harboring *ProMGD3*:*DGAT2, DGAT2* expression in *DGAT2*_WT line 6 was ~6-fold higher than that in WT and *pgm-1* plants under Pi-depleted conditions, whereas expression under Pi-sufficient conditions was similar to that of WT and *pgm-1* plants (Figure 4B). Among three lines of *DGAT2_pgm-1* transgenic plants, *DGAT2* expression levels in lines 2 and 3 were only slightly higher than that in WT and *pgm-1* plants under Pi-depleted conditions (~2.5-fold higher; Figure 4B). *PDAT1* expression levels in *PDAT1*_WT line 4 and *PDAT1_pgm-1* line 3 were ~2.5-fold higher under Pi-sufficient conditions and markedly higher under Pi-depleted conditions (~50− and ~80-fold higher, respectively) compared with WT and *pgm-1* plants (Figure 4C).

**Figure 5:**
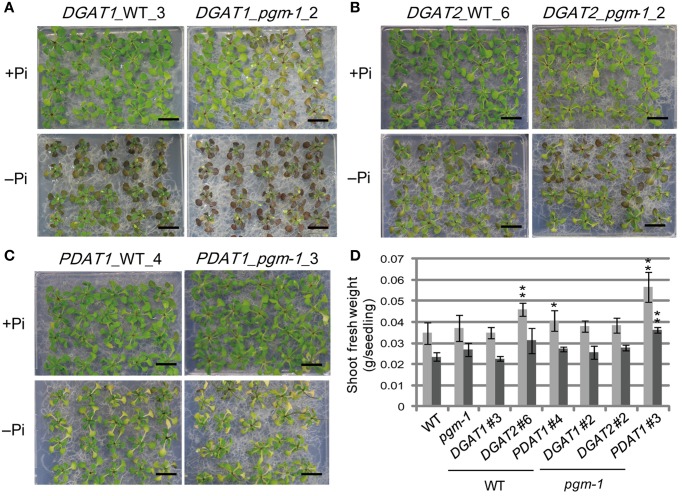
**Growth phenotypes and fresh weight of transgenic lines under Pi-sufficient and Pi-depleted conditions**. Seedlings (10 d old) of transgenic lines were transferred to MS agar containing 1% (w/v) sucrose and 0 mM (–Pi) or 1 mM (+Pi) Pi and were grown for 10 d. Growth phenotypes of **(A)** *DGAT1*_WT_3 and *DGAT1*_*pgm-1*_2, **(B)** *DGAT2*_WT_6 and *DGAT2_pgm-1*_2, and **(C)** *PDAT1*_WT_4 and *PDAT1*_*pgm-1*_3. Bars = 1 cm. **(D)** Shoot fresh weights of seedlings grown under Pi-sufficient (light gray) and Pi-depleted (dark gray) conditions. Data are the mean ± SD from three independent experinments; ^*^P < 0.05 or ^**^P < 0.01 for *t*-test vs. WT under Pi-sufficient conditions.

Under both Pi conditions, growth phenotypes and shoot fresh weights of *DGAT1*_WT line 3 and *DGAT1_pgm-1* line 2 were similar to those of WT and *pgm-1* plants (Figures 3A, 5A,D). *DGAT2*_WT line 6 and *DGAT2_pgm-1* line 2 accumulated slightly less anthocyanin than did WT and *pgm-1* plants under both Pi conditions (Figures 3A, 5B,D). The shoot fresh weight of *DGAT2*_WT line 6 grown under Pi-sufficient conditions was greater than those of WT and *pgm-1* plants, although under Pi-depleted conditions the fresh weight was similar to those of WT and *pgm-1* plants (Figure 5D). The growth phenotype of *PDAT1*_WT line 4 was similar to that of *PDAT1_pgm-1* line 3 under both Pi conditions but differed from those of WT and *pgm-1* plants (Figures 3A, 5C). Under Pi-depleted conditions, seedlings of both lines were yellowish and accumulated markedly less anthocyanin than did WT and *pgm-1* plants (Figures 3A, 5C). Moreover, the fresh weight of *PDAT1_pgm-1* line 3 was significantly greater than those of WT and *pgm-1* plants under both Pi conditions (Figure 5D). Taken together, the shoot fresh weights of all of the transgenic plants was similar to, or higher than, those of WT and *pgm-1* plants under both Pi conditions (Figure 5D).

### Pi starvation–induced overexpression of *DGAT1, DGAT2*, and *PDAT1* enhances TAG accumulation in vegetative tissues

**Figure 6:**
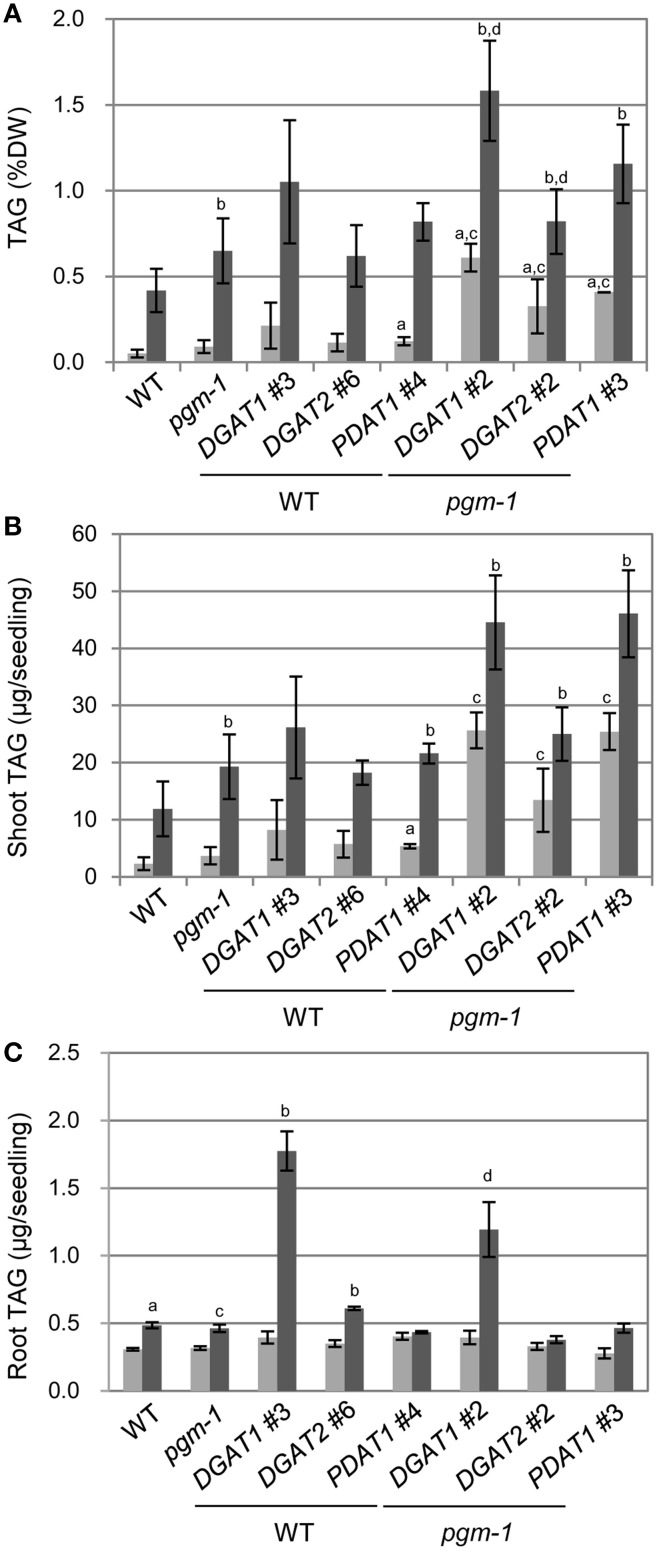
**TAG accumulation in *Arabidopsis* transgenic lines under Pi-sufficient and Pi-depleted conditions**. Plants were grown on MS agar with 1% (w/v) sucrose for 10 d and then were transferred to MS agar containing 1% (w/v) sucrose with 1 mM Pi or without Pi for 10 d. **(A)** TAG levels in shoots per dry weight (DW) in WT, *pgm-1*, and transgenic plant lines under Pi-sufficient (light gray) and Pi-depleted (dark gray) conditions. **(B**,**C)** TAG content in shoots **(B)** and roots **(C)** per each seedling of WT, *pgm-1*, and transgenic plant lines, as in **(A)**. Data are the mean ± SD from three independent experiments; ^*a*−*d*^*t*-test significant at *P* < 0.05 vs. ^*a*^WT under Pi-sufficient conditions, ^*b*^WT under Pi-depleted conditions, ^*c*^*pgm-1* under Pi-sufficient conditions, or ^*d*^*pgm-1* under Pi-depleted conditions.

We analyzed TAG levels in vegetative tissues of WT, *pgm-1*, and transgenic plants (Figure 6). Compared with WT plants under Pi-sufficient conditions, TAG levels in *pgm-1* under Pi-sufficient and Pi-depleted conditions were 1.8-fold and 13-fold higher, respectively (Figure 6A). Moreover, TAG levels in *DGAT1_pgm-1* line 2, *DGAT2_pgm-1* line 2, and *PDAT1_pgm-1* line 3 transgenic lines under Pi-depleted conditions were 26-,16-, and 23-fold higher, respectively, than WT under Pi-sufficient conditions (Figure 6A). Moreover, the TAG content in shoots per seedling of *pgm-1, DGAT1_pgm-1* line 2, *DGAT2_pgm-1* line 2, and *PDAT1_pgm-1* line 3 transgenic lines under Pi-depleted conditions was 9.5-, 19-, 12-, and 23-fold higher, respectively, than that of WT seedlings under Pi-sufficient conditions, and the TAG content was 1.8-, 3.5-, 2.3-, and 4.3-fold higher, respectively, than that of WT seedlings under Pi-depleted conditions (Figure 6B).

We also measured the TAG content in roots per seedling in six transgenic lines under Pi-sufficient and Pi-depleted conditions (Figure 6C). The overexpression of these three genes had different effects in roots than in shoots. First, the TAG content in roots per seedling of WT and *pgm-1* was comparable under both Pi conditions (Figure 6C). Second, *DGAT1* overexpression in WT and *pgm-1* plants was the most effective way to produce more TAGs under Pi-depleted conditions compared with *DGAT2*- or *PDAT1*-containing transgenic plants in the same background (Figure 6C). Under Pi-depleted conditions, the TAG content in *DGAT1*_WT line 3 and *DGAT1_pgm-1* line 2 increased to 5.8-fold and 4-fold of the levels in WT and *pgm-1*, respectively (Figure 6C). However, in the other transgenic plants, the TAG content was comparable with that in the non-transformed backgrounds, WT and *pgm-1* (Figure 6C). Thus, using the starchless mutant background during Pi starvation in combination with the overexpression of *DGAT1, DGAT2*, or *PDAT1* was the most efficient way to increase TAG levels in shoots (Figures 6A,B), but for roots, the use of the *pgm-1* background for transgenic plants, relative to WT, had a negative effect on increasing TAG levels. Although, the level of TAG in *DGAT1_pgm-1* roots increased by 4-fold under Pi-depleted conditions compared with that in *pgm-1* under Pi-sufficient conditions, the TAG level in the roots of *pgm-1* and the other *pgm-1* background transgenic plants was similar to, or smaller than, that in WT and the other WT background transgenic plants (Figure 6C). Thus, our results clearly showed that the Pi starvation–induced overexpression of *DGAT1* or *PDAT1* in the *pgm-1* mutant background is the most efficient way to increase TAG levels in shoots, but *DGAT1* overexpression in WT is effective for enhancing TAG accumulation in roots.

## Discussion

Plants increase TAG levels in vegetative tissues during senescence and under several stresses such as freezing, drought stress, or oxidative stress (Sakaki et al., 1990a,b,c; Kaup et al., 2002; Gaude et al., 2007; Moellering et al., 2010; Lippold et al., 2012; Troncoso-Ponce et al., 2013). Among the essential macronutrients for plants, N starvation is a well-known trigger for TAG synthesis in leaves (Gaude et al., 2007; Yang et al., 2011; Lippold et al., 2012). However, N starvation promotes senescence and degradation of chlorophyll, causing a severe reduction in photosynthetic activity, and thus results in smaller seedlings as compared with well-fertilized plants (Yang et al., 2011). We showed that Pi starvation alters TAG levels in seedlings and compared the effect with N starvation (Figures 1, 2). Although, N starvation also results in TAG accumulation, the biomass of plants grown under N-depleted conditions was markedly smaller than that under Pi-depleted conditions (Figure 1C). Moreover, chloroplast ultrastructure was not severely affected, during Pi deficiency except for an increase in the number of starch granules in the stroma (Supplementary Figure 1). Taken together, these results suggest that either N or Pi starvation results in TAG accumulation in leaves, but the damage to plants is relatively more severe during N starvation. Indeed, *Arabidopsis* WT plants under Pi-depleted conditions maintain ~85% of their photosynthetic activity as compared with plants grown under Pi-sufficient conditions (Kobayashi et al., 2009). These results also suggest that both types of nutrient deficiency result in TAG accumulation in leaves, but the pathways and the mechanisms of TAG synthesis might differ slightly.

Because, TAG levels were increased in both shoots and roots, we first thought that the TAG synthesis genes were up-regulated under Pi-depleted conditions. Unexpectedly, the expression levels of three major TAG synthesis genes, *DGAT1, DGAT2*, and *PDAT1*, were not significantly increased in WT shoots under Pi-depleted conditions (Figures 4A–C). Moreover, in WT roots, although expression levels of *DGAT2* remained unchanged under both Pi conditions, those of *DGAT1* and *PDAT1* under Pi-depleted conditions were significantly decreased compared with those under Pi-sufficient conditions (Figure 4D). These results suggested that enhanced TAG accumulation under Pi-depleted conditions was not correlated with the transcriptional up-regulation of TAG synthesis genes, as was also suggested by Pant et al. (2015), but could be due to the down-regulation of genes involved in TAG breakdown or homeostasis during Pi depletion (James et al., 2010; Kelly et al., 2013; Park et al., 2013). Currently, the molecular mechanisms behind Pi starvation**–**induced TAG accumulation are unclear but are under investigation.

We clearly showed that the Pi starvation–inducible promoter *ProMGD3* is a useful engineering tool for producing transgenic plants that accumulate TAG in shoots in response to Pi starvation; however, it was only partially successful in roots. As the roots of *DGAT1*_WT accumulated significant levels of TAG in response to Pi starvation (Figure 6C), the reason for unchanged TAG levels in *DGAT2*- or *PDAT1***-**containing transgenic plants might be due to the decreased availability of the preferred substrates in roots compared with shoots under Pi-depleted conditions. Indeed, the substrate preferences of *Arabidopsis DGAT1* and *DGAT2* were reported to be different (Zhou et al., 2013; Ayme et al., 2014). As for the fatty acid composition of TAGs in roots of WT, *pgm-1*, and transgenic plants under Pi-depleted conditions, both *DGAT1*_WT and *DGAT1*_*pgm-1* showed notable increases in the 18:1 ratio and decreases in the 18:3 ratio compared with those in the other plants (Supplementary Figure 3). However, for the fatty acid composition of TAGs in shoots, an increase in the 18:1 ratio and a decrease in the 18:3 ratio were observed only in *DGAT1*-containing transgenic plants under Pi-depleted conditions (Supplementary Figure 4). The fatty acid composition of TAGs in *DGAT1*-containing transgenic plants was in agreement with the substrate preference, 18:1 over 18:3, of DGAT1 (Zhou et al., 2013). Thus, these transgenic plants might also be useful for further analyzing the differences in TAG accumulation mechanisms and the availability of substrates in shoots and roots under Pi starvation.

In this study, we enhanced TAG accumulation in vegetative tissues using the promoter of a plastid-localized galactolipid synthase gene in combination with the lipid remodeling system under Pi starvation, but the amount of TAG was still low compared with the levels seen in previous studies. The highest TAG levels in shoot of our transgenic plants was ~1.5 % D.W. as shown in Figure 6A. Fan et al. (2013) showed that coexpression of PDAT1 with oleosin in wild-type background and *tgd1-1* mutant background boost leaf TAG content by up to 6.4–8.6% of the dry weight, respectively. Kelly et al. (2013) showed that in transgenic plants constitutively coexpressing WRINKLED1 and DGAT1 in *sdp1* mutant background, the accumulation of TAG in roots, stems, and leaves was elevated to levels ranging from 5 to 8% of dry weight. Thus, all the other works were performed by engineering multiple genes involved in TAG accumulation or degradation such as co-overexpression of oleosin or knock-out of *SDP1*. Given that we only introduced single gene into the transgenic plants, we still have chance to elevate TAG levels in leaves by engineering multiple genes using our system. Moreover, we are currently investigating whether this system can be applied to crop plants, and we are determining the best soil growth conditions for enhanced TAG accumulation without extreme plant growth retardation. Although, we showed its ability to produce TAG only in vegetative tissues, this system can be applied to producing other useful industrial compounds by introducing the corresponding synthesis genes in place of the TAG synthesis genes. Thus, we hope that the system will be used in many industrial applications in the near future.

## Materials and methods

### Plant material and growth conditions

Seeds of *pgm-1* were obtained from the Arabidopsis Biological Resource Center. Surface-sterilized seeds of WT *A. thaliana* (Columbia-0), the starchless mutant *pgm-1*, and transgenic mutant lines were incubated at 4°C in darkness for 3 d prior to plating on Murashige and Skoog (MS) medium (Murashige and Skoog, 1962) containing 0.8% (w/v) agar (MS agar) supplemented with 1% (w/v) sucrose and were then incubated at 23°C under continuous white light (40–50 μmol m^−2^ s^−1^) for all growth conditions. *Arabidopsis*, tomato (*S. lycopersicum* L.), barnyard grass (*E. crus-galli*), and tobacco (*N. tabacum*) seeds were grown on solidified MS agar supplemented with 1% (w/v) sucrose for 8 d (tomato, barnyard grass) or 10 d (*Arabidopsis* and tobacco) and then were grown for another 10 d (*Arabidopsis*), 21 d (tobacco), or 28 d (tomato and barnyard grass) on solidified Pi-sufficient (1 mM Pi) or Pi-depleted (0 mM Pi) medium (Härtel et al., 2000) supplemented with 1% (w/v) sucrose, or for 7 d on solidified N-sufficient (3.5 mM N) or N-depleted (0 mM N) medium supplemented with 1% (w/v) sucrose, with KNO_3_ and Ca(NO_3_)_2_ 4H_2_O replaced with KCl_2_ and CaCl_2_, respectively.

### Electron microscopy

Leaf segments were fixed with 2% (w/v) paraformaldehyde and 2.5% (w/v) glutaraldehyde in 0.067 M sodium phosphate buffer (pH 7.4) for 2 h at room temperature and then for 16 h at 4°C. Samples were then washed six times in the sodium phosphate buffer for 10 min each at room temperature. They were post-fixed with 2% (w/v) osmium tetroxide in 0.067 M sodium phosphate (pH 7.4) for 2 h at room temperature. The fixed samples were dehydrated in a graded ethanol series and embedded in epoxy resin mixture (Quetol 651 mixture; Nissin EM). Ultrathin 70-nm sections were cut with a diamond knife on a Leica Ultracut UCT ultramicrotome and were transferred onto copper grids. The sections were stained with 2% (w/v) uranyl acetate for 15 min followed by 0.4% (w/v) lead citrate for 5 min at room temperature. The specimens were observed on a Hitachi H-7500 transmission electron microscope at an accelerating voltage of 80 kV.

### Lipid analysis

Total lipid was extracted from tissues as described by Bligh and Dyer (1959). The polar membrane lipids were separated by two-dimensional thin-layer chromatography (Kobayashi et al., 2007). TAGs were separated by one-dimensional thin-layer chromatography using the solvent system of hexane/diethyl ether/acetic acid (160:40:4, v/v/v). Lipids on silica gel plates were visualized with 0.01% (w/v) primuline in 80% (v/v) acetone under UV light. Lipids isolated from silica gel plates were methylated, and fatty acid methyl esters were quantified by gas chromatography using pentadecanoic acid as an internal standard (Kobayashi et al., 2006).

### Imaging lipid droplets *in situ*

Leaves were vacuum-fixed in 4% (w/v) paraformaldehyde in 50 mM PIPES buffer (pH 7.0) and stained with 50 μg ml^−1^ Nile red (Sigma) to selectively visualize lipid droplets *in situ* (Greenspan et al., 1985). The Nile Red signal and chlorophyll autofluorescence were observed using a confocal laser-scanning microscope (TCS SE; Leica) with an argon laser for excitation at 488 nm, a 556- to 580-nm filter for detection of the Nile Red signal, and a 718- to 749-nm filter for detection of chlorophyll fluorescence. Images were merged and pseudocolored using Leica confocal software.

### Generation and selection of transgenic plants

To obtain transgenic plants, the complete coding sequences of *DGAT1, DGAT2*, and *PDAT1* from *A. thaliana* were amplified from the reverse transcript of WT leaf total RNA. The primers used were DGAT1_FW (5′ CGCCCGGGTATGGCGATTTTGGATTCTGCTGGC 3′), DGAT1_RV (5′ GCGAGCTCTCATGACATCGATCCTTTTCGGTTC 3′); DGAT2_FW (5′ GCCCCGGGTATGGGTGGTTCCAGAGAGTTCCGAG 3′), DGAT2_RV (5′ GCGAGCTCTCAAAGAATTTTCAGCTCAAGATC 3′); and PDAT1_FW (5′ CGCCCGGGTATGCCCCTTATCATCGGAAAAAG 3′), PDAT1_RV (5′ GCGAGCTCTCACAGCTTCAGGTCAATACGCTC 3′). Each amplified fragment was cloned into the pZErO cloning vector (Life Technologies). To remove the *Sac* I site in the *DGAT1* coding sequence, the obtained vector was subjected to a Quikchange Lightning reaction (Qiagen) using the following primers: DGAT1_c845t_fw (5′ GTCTCCTACTACGTTAGCTTGAAGAGCTTGGCATATTTC 3′) and DGAT1_c845t_rv (5′ GAAATATGCCAAGCTCTTCAAGCTAACGTAG TAGGAGAC3′). Vectors were subjected to restriction analysis and DNA sequencing to confirm the presence of the expected sequences. Each *DGAT1, DGAT2*, and *PDAT1* fragment was digested with *Sma* I and *Sac* I and independently ligated into the *Sma* I and *Sac* I sites of plasmid *atMGD3*::GUS/pBI101 (Kobayashi et al., 2004). All of the *Arabidopsis* transformants described here were produced using a modified version of the floral dip method (Clough and Bent, 1998) and were selected on MS agar containing 50 μg ml^−1^ kanamycin.

### Quantitative reverse transcription–PCR

Total RNA was isolated from three independent plant samples using the SV Total RNA Isolation System (Promega). Reverse transcription was performed using the PrimeScript RT reagent kit (TaKaRa Bio). cDNA amplification was carried out using SYBR PreMix Ex Taq (TaKaRa Bio). Signal detection and quantification were performed in duplicate using the Thermal Cycler Dice Real Time System (TaKaRa Bio). Quantitative PCR determination of *DGAT1, DGAT2*, and *PDAT1* transcripts was carried out using the *Arabidopsis UBQ10* transcript levels for normalization (Narise et al., 2010). Expression levels were obtained from at least three replicates. The gene-specific primers used were as follows: DGAT1_fw (5′ GAGAGAGAGTCCA CTTAGCTC 3′), DGAT1_rv (5′ CGTTCTGATCAAC CAACCATAC 3′); DGAT2_fw (5′ TCCAGCCTAATCG TGCCTATG 3′), DGAT2_rv (5′ GGGAGTGTAGAAT ATAGCACTAC 3′); PDAT1_fw (5′ AGGCAAACAATGCGCT GATGG 3′), PDAT1_rv (5′ TGTCAAGTGACAT GTGTTCCAC 3′); UBQ10_fw (5′ GGCCTTGTATAATCCC TGATGAATAAG 3′), UBQ10_rv (5′ AAAGAGATAACAGGA ACGGAAACATAGT 3′).

### Conflict of interest statement

The authors declare that the research was conducted in the absence of any commercial or financial relationships that could be construed as a potential conflict of interest.

## Supplementary material

Supplementary Figure 1 **Electron microscopy of leaf mesophyll cells in WT *Arabidopsis* plants**. Plants were grown on MS agar with 1% (w/v) sucrose for 10 d and then were transferred to MS agar containing 1% (w/v) sucrose without (0 mM) Pi for 10 d. White arrows indicate oil droplets. S, starch; M, mitochondrion. Bars = **(A)** 10 μm and **(B)** 0.5 μm.

Supplementary Figure 2 **TAG levels in various plants under Pi-depleted conditions**. **(A,B)** TAG levels in tomato (*S. lycopersicum L*.) shoots **(A)** and barnyard grass (*E. crus-galli*) **(B)** grown for 13 d with Pi and then for 31 d with (+) or without (–) Pi. For all conditions: +Pi, 1 mM; –Pi, 0 mM. **(C,D)** Fatty acid profiles of TAGs in shoots of tomato **(C)** and barnyard grass **(D)**. Data are the mean ± SD from three independent experiments.

Supplementary Figure 3 **Fatty acid composition of TAG in roots of WT, *pgm-1*, and transgenic plant lines grown under Pi-sufficient and Pi-depleted conditions**. Plants were grown on MS agar with 1% (w/v) sucrose for 10 d and then were transferred to MS agar containing 1% (w/v) sucrose with **(A)** or without **(B)** Pi for 10 d. Data are the mean ± SD from three independent experiments.

Supplementary Figure 4 **Fatty acid composition of TAG in shoots of WT, *pgm-1*, and transgenic plant lines**. Plants were grown under Pi-sufficient (gray) and Pi-depleted (black) conditions in all of the figures, except the WT (N) figure, in which plants were grown under N-sufficient (gray) and N-depleted (black) conditions. Data are the mean ± SD from three independent experiments.
