# Supplementary figures and images for: An engineered lipid remodeling system using a galactolipid synthase promoter during phosphate starvation enhances oil accumulation in plants
Source: Front Plant Sci. 2015 Aug 31;6:664. doi: 10.3389/fpls.2015.00664 (PMC4553410; doi:10.3389/fpls.2015.00664)

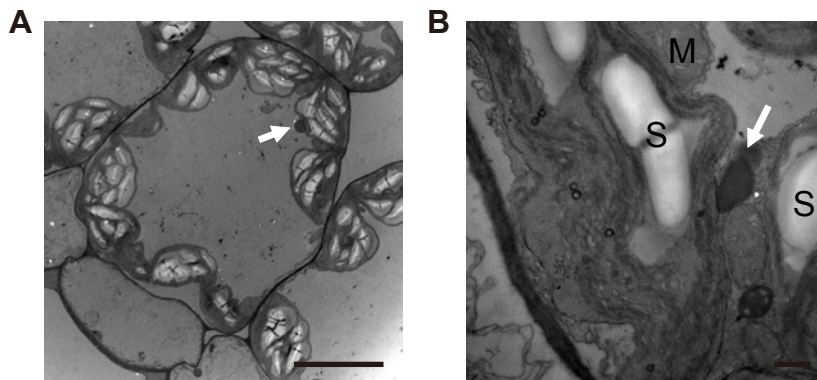

Supplementary Figure 1 Shimojima et al. FPS

Supplement: Supplementary Figure 1 — Electron microscopy of leaf mesophyll cells in WT Arabidopsis plants. Plants were grown on MS agar with 1% (w/v) sucrose for 10 d and then were transferred to MS agar containing 1% (w/v) sucrose without (0 mM) Pi for 10 d. White arrows indicate oil droplets. S, starch; M, mitochondrion. Bars = (A) 10 μm and (B) 0.5 μm. [file Image1.PDF]

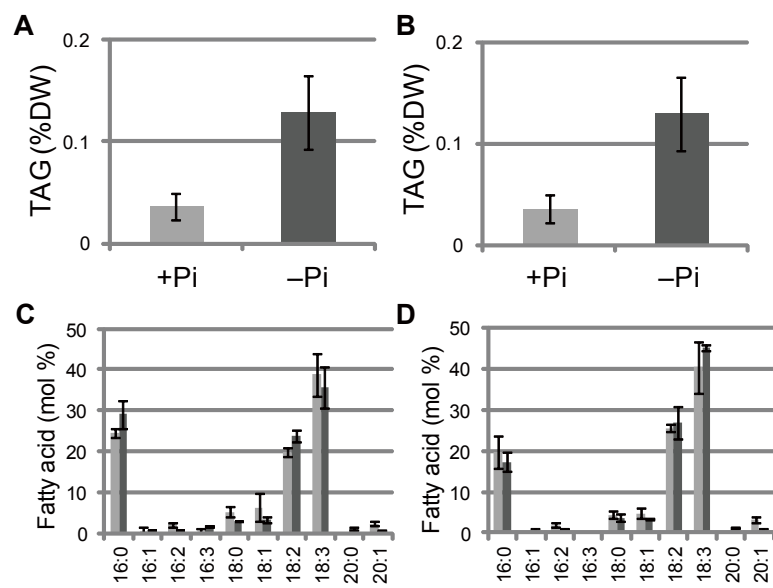

Supplementary Figure 2 Shimojima et al. FPS

Supplement: Supplementary Figure 2 — TAG levels in various plants under Pi-depleted conditions. (A,B) TAG levels in tomato (S. lycopersicum L.) shoots (A) and barnyard grass (E. crus-galli) (B) grown for 13 d with Pi and then for 31 d with (+) or without (–) Pi. For all conditions: +Pi, 1 mM; –Pi, 0 mM. (C,D) Fatty acid profiles of TAGs in shoots of tomato (C) and barnyard grass (D). Data are the mean ± SD from three independent experiments. [file Image2.PDF]

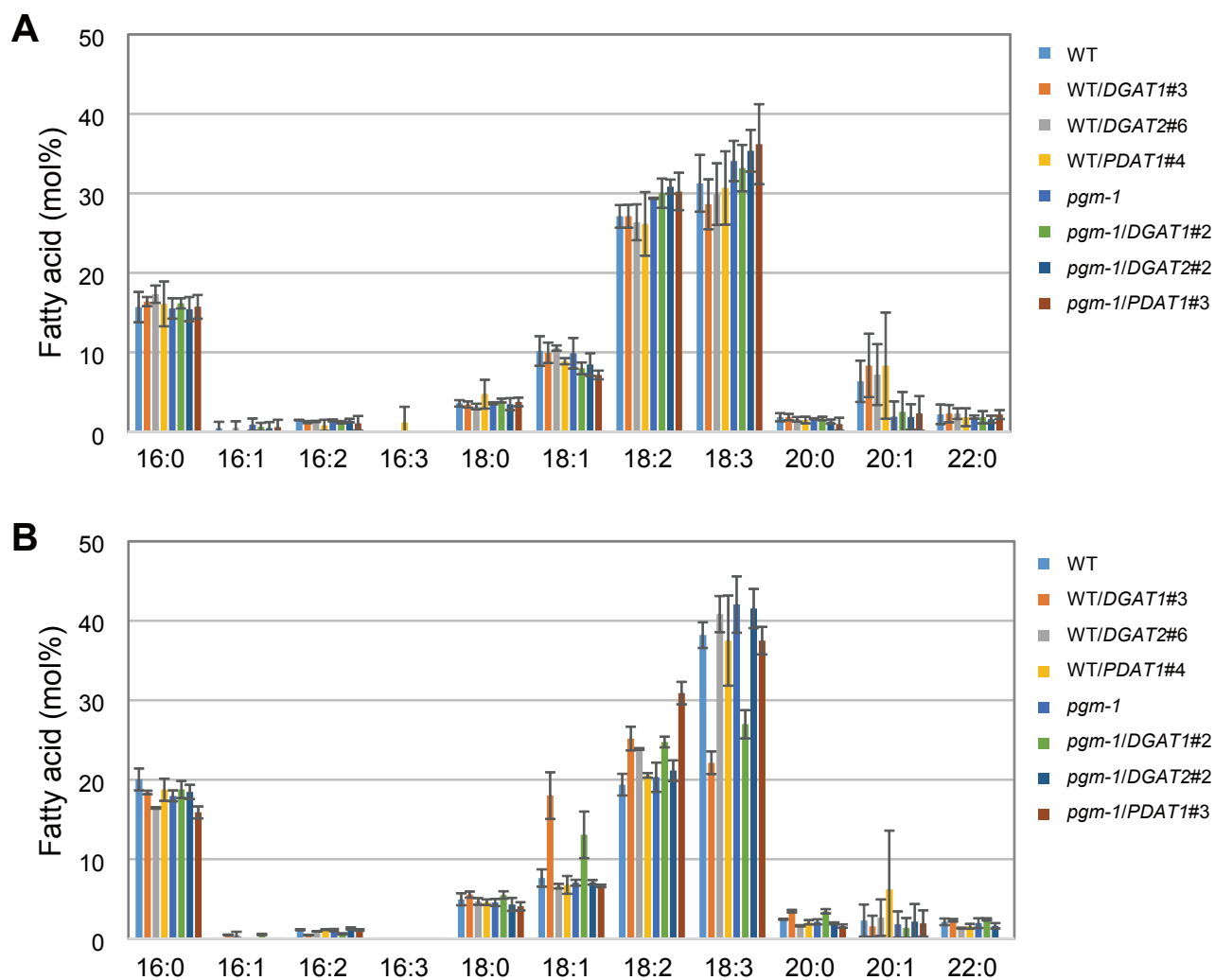

Supplementary Figure 3 Shimojima et al. FPS

Supplement: Supplementary Figure 3 — Fatty acid composition of TAG in roots of WT, pgm-1, and transgenic plant lines grown under Pi-sufficient and Pi-depleted conditions. Plants were grown on MS agar with 1% (w/v) sucrose for 10 d and then were transferred to MS agar containing 1% (w/v) sucrose with (A) or without (B) Pi for 10 d. Data are the mean ± SD from three independent experiments. [file Image3.PDF]

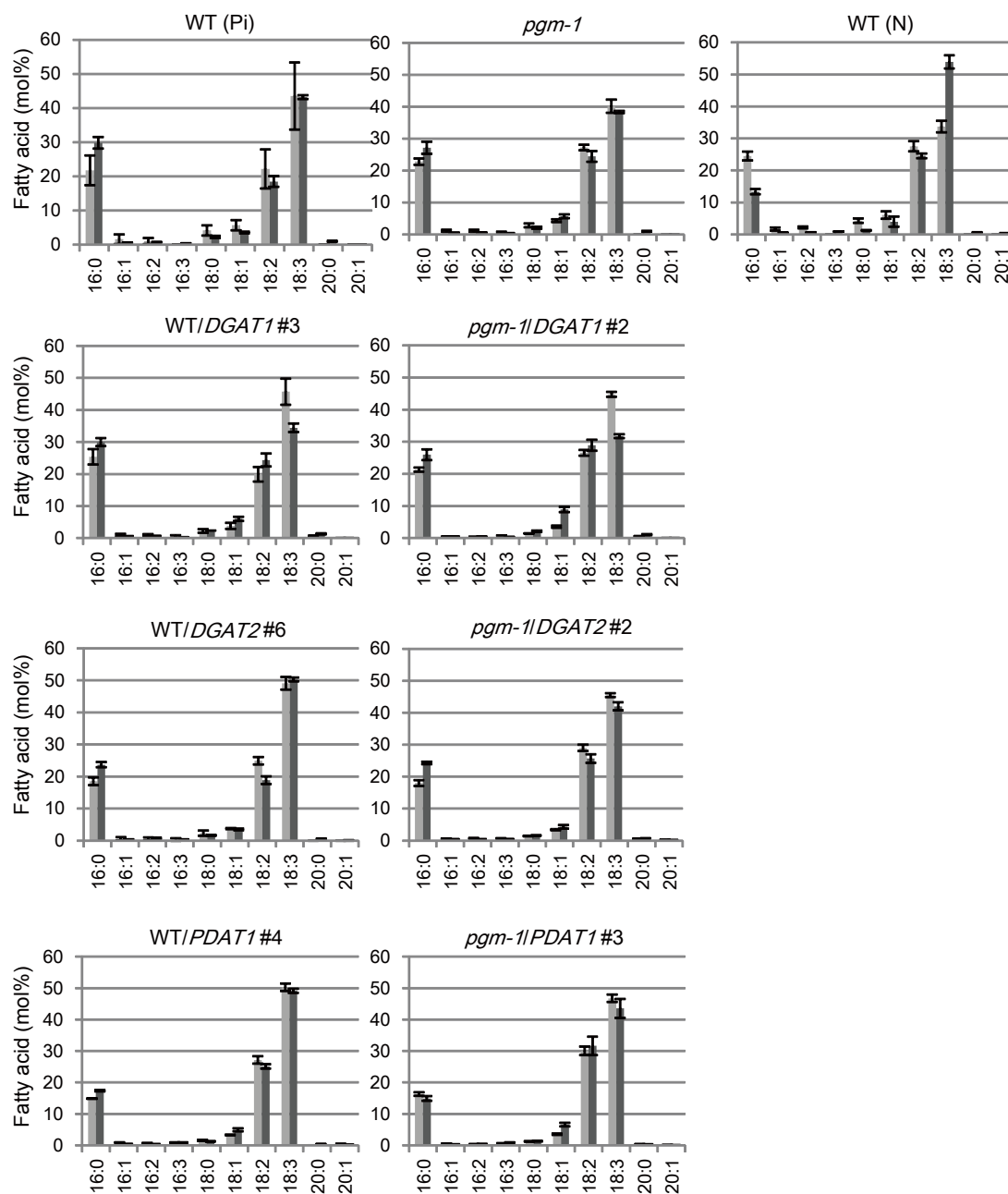

Supplementary Figure 4 Shimojima et al. FPS

Supplement: Supplementary Figure 4 — Fatty acid composition of TAG in shoots of WT, pgm-1, and transgenic plant lines. Plants were grown under Pi-sufficient (gray) and Pi-depleted (black) conditions in all of the figures, except the WT (N) figure, in which plants were grown under N-sufficient (gray) and N-depleted (black) conditions. Data are the mean ± SD from three independent experiments. [file Image4.PDF]
